# CAR T Cell Therapy for Chronic Lymphocytic Leukemia: Successes and Shortcomings

**DOI:** 10.3390/curroncol29050293

**Published:** 2022-05-18

**Authors:** Zeljko Todorovic, Dusan Todorovic, Vladimir Markovic, Nevena Ladjevac, Natasa Zdravkovic, Predrag Djurdjevic, Nebojsa Arsenijevic, Marija Milovanovic, Aleksandar Arsenijevic, Jelena Milovanovic

**Affiliations:** 1 Department of Internal Medicine, Faculty of Medical Sciences, University of Kragujevac, 34000 Kragujevac, Serbia; todorovic_zeljko@hotmail.com (Z.T.); natasasilvester@gmail.com (N.Z.); pdjurdjevic@sbb.rs (P.D.); 2 Department of Ophthalmology, Faculty of Medical Sciences, University of Kragujevac, 34000 Kragujevac, Serbia; drdusantodorovic@yahoo.com; 3 Center for Molecular Medicine and Stem Cell Research, Faculty of Medical Sciences, University of Kragujevac, 34000 Kragujevac, Serbia; vladimirmarkovic.vlad@gmail.com (V.M.); dr.nevena.ladjevac@gmail.com (N.L.); arne@medf.kg.ac.rs (N.A.); marijaposta@gmail.com (M.M.); 4 Department of Histology and Embryology, Faculty of Medical Sciences, University of Kragujevac, 34000 Kragujevac, Serbia

**Keywords:** CAR T cells, CAR NK cells, CLL

## Abstract

Chimeric antigen receptor T (CAR T) cell therapy achieved remarkable success in B-cell leukemia and lymphoma which led to its incorporation in treatment protocols for these diseases. CAR T cell therapy for chronic lymphocytic leukemia (CLL) patients showed less success compared to other malignant tumors. In this review, we discuss the published results regarding CAR T cell therapy of CLL, possible mechanisms of failures and expected developments.

## 1. Introduction

Chronic lymphocytic leukemia (CLL) is a chronic lymphoproliferative disease characterized by malignant transformation of mature antigen-experienced B lymphocyte and accumulation of monoclonal malignant B cells in peripheral blood, bone marrow, lymph nodes, spleen [1]. CLL is the most common leukemia in Western countries. The incidence of CLL in North America and Eastern Europe is 4.7 per 100,000 people per year, while the CLL incidence is higher than 35 in the population over 85 years of age [2]. Clinical management of CLL is challenging and depends on patients ages, comorbidities and biological features of CLL cells such as immunoglobulin heavy chain gene mutation, 17p deletion, TP53 mutation and, as recent research showed, number and type of CLL tumor clones in patient [3,4]. Treatment option varies from watch and wait approach in the asymptomatic early-stage CLL to chemo-immunotherapy and novel targeted therapies, such as Bruton’s tyrosine kinase inhibitors and inhibitors of Bcl-2 family proteins, for symptomatic and advanced disease [3]. However, despite the expansion of novel therapeutic approaches, CLL is still mostly an incurable disease.

**Figure 1:**
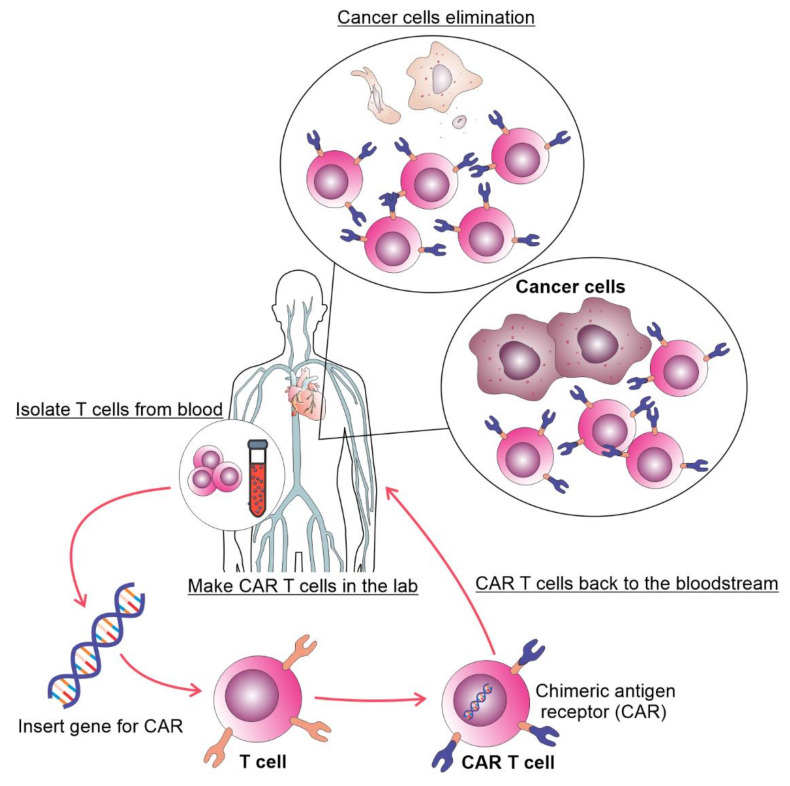
The process of autologous CAR T cell therapy. T cells are collected via apheresis, genetically reengineered in a laboratory by introducing DNA that encodes CAR, multiplied, and then infused into the patient. The CAR T cells may eliminate all of the cancer cells and may remain in the body months after the infusion.

In the last decades, cell-based immunotherapy has emerged as a novel treatment for malignant diseases. Cell based immunotherapy relies on using immune cells obtained from patients, raised in vitro, and genetically modified to increase their ability to find and kill tumor cells (Figure 1). Various T cell based treatments of malignant disorders have been developed, including tumor-infiltrating lymphocytes, T cell receptor (TCR)-modified T cells and chimeric antigen receptor T (CAR T) cells [5]. Therapy of malignancies with CAR T cells is accompanied with better and durable clinical responses compared to the treatment with tumor-infiltrating lymphocytes and TCR modified T cells [6,7]. CAR T cells are T lymphocytes with engineered synthetic receptors made to recognize and destroy the cells expressing the target antigen. CARs are artificially made proteins consisting of the single-chain variable fragment of an antibody (ScFv) that recognizes tumor antigen and the T-cell activation domain [8].

**Figure 2:**
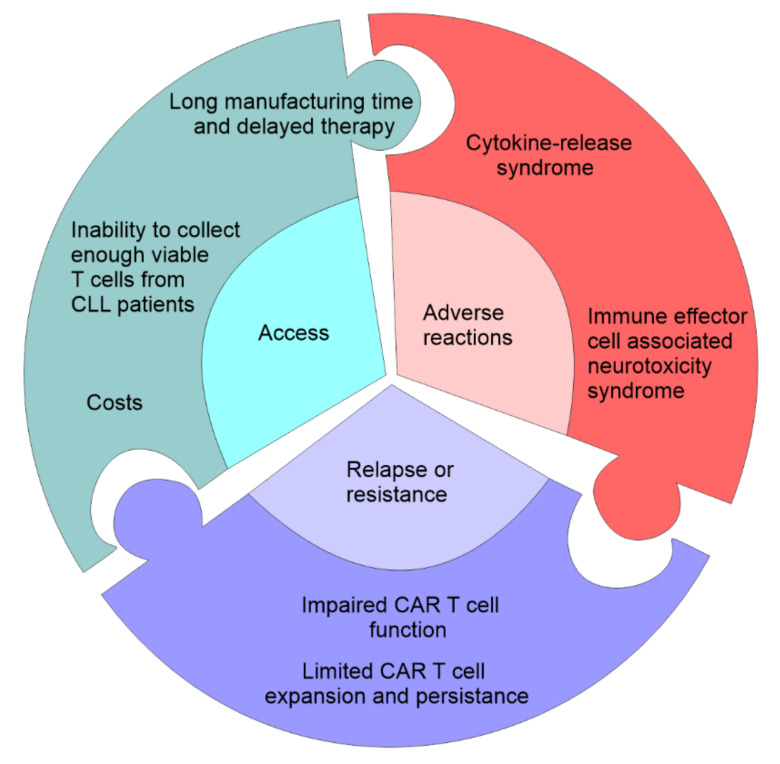
Challenges in CAR T-cell therapy. The first problem associated with CAR T cell therapy is access which can be limited by the cost of the manufacture of CAR T cells, time needed to manufacture and expand CAR T cells, failure to produce autologous CAR T cells, and eligibility for the clinical trial. The second problem associated with CAR T cell therapy are serious adverse event: cytokine-release storm (widespread pyroptosis of tumor cells is followed by the release of different factors that activate macrophages to produce inflammatory cytokines that induce systemic inflammatory response), and neurotoxicity syndrome (toxic encephalopathy caused by the disruption of the blood-brain-barrier). CAR T cell therapy may be accompanied by the primary resistance due to the impaired CAR T cell function and the inability to induce remission and limited CAR T cell expansion.

According to the T-cell activation domain, which is part of the hybrid receptor, CAR T cells are classified into four generations. The first generation of CAR T cells contains only CD3 zeta domain in fusion with receptors specific for a specified tumor antigen. These first generation CAR T cells showed limited clinical effects. Hybrid receptors of the second generation of CAR T cells in addition to CD3 contain one of the additional costimulatory molecules such as CD28, ICOS, CD-137/4-1BB or OX40, while coupling two or more costimulatory molecules into the receptor sequence led to the creation of the third generation CAR T cells [8]. The fourth CAR T cells’ generation is engineered with the inducible expression of cytokines that potentiate antitumor immunity and a self-withdrawal mechanism, with a suicide gene that can be activated after the achievement of the anti-tumor effect whose product rapidly withdraws CAR T cells [9]. The good clinical responses to CAR T cell therapy in some malignancies are frequently accompanied by several obstacles (Figure 2). Since most of the patients are highly medicated resulting in a low number and quality of T cells, manufacturing and expanding autologous CAR T cells from such patients can be difficult [10]. The manufacture of allogenic CAR T cells from healthy donors is promising but allogenic CAR T cells can cause a serious graft-versus-host disease (GvHD) [11]. However, the fact that allogenic NK cells have reduced the risk for induction of GvDH led to the construction of CAR Natural Killer (NK) cells. CAR constructs in CAR NK cells play the role in the NK cell activation and also improve the efficiency of innate ability of NK cells to kill malignant cells [12].

Although the use of cellular immunotherapy in many hematologic malignancies is already approved outside clinical trials [13,14,15,16], the benefit of CAR T and CAR NK cells in CLL treatment is still not clear. In this review, we summarize existing data regarding results of CAR T and CAR NK therapy of CLL, associated difficulties, and potential strategies to overcome them.

## 2. CAR-T as Promising CLL Therapy

In the early stage of development of CAR-T cells for B cell leukemia, CD19 has become attractive target antigen because it is expressed on the most B-cell malignancies [17]. Results of preclinical studies indicate better in vivo expansion and far greater effectiveness of the second generation of CAR-T cells, with CARs constructed to target CD19 coupled with CD137 signaling or CD28 costimulatory domain, in comparison to the first generation of CAR T cells [18,19]. The first use of CAR T cells in the therapy of CLL was reported in 2011 [20]. Porter et al. reported the treatment of two patients with refractory/relapsed CLL with reinfusion of approximately 1.5 × 10^5^ autologous CAR T cells per kilogram of body weight that expanded to a level that was more than 1000 times higher than the initial engraftment level in vivo [20]. After treatment, patients achieved complete remission (CR) [20]. Further, it has been recently reported that both patients sustained remission for more than ten years after the therapy and that CAR T cells are still detectable in their blood [21]. These long-persisting CAR T cells are CD4+ cells with cytotoxic capabilities, but also an expanded population of gamma delta CAR T cells has been found in one patient concomitant with CD8+ CAR T cells during the initial response phase [21].

So far, over 100 CLL patients have been treated with anti-CD19 CAR-T cells (Table 1). The majority of studies included relapsed patients or patients refractory to conventional therapy regimens, except for one which enrolled patients with only a partial response (PR) to the first line therapy [22]. Overall response rate (ORR; sum of CR and PR) varies between studies. Brentjens et al. suggested that chemotherapy that induced the depletion of lymphocytes prior to CAR T infusion enhanced the efficacy of CAR T cells [23]. In line with this are the results of the studies reporting the lowest ORR in patients without lymphodepletion therapy prior to CAR T infusion [23,24,25,26]. It is considered that lymphodepletion chemotherapy reduced tumor mass but also the number of regulatory cells, which may attenuate the antitumor activity of the infused CAR T cells.

Another factor that could influence response to therapy is a number of CLL T applied to patients. One recent study has shown that a higher dose of anti-CD19 CAR T cells (5.0 × 10^8^ vs. 5.0 × 10^7^) produces higher rates of ORR (55% vs. 31%) and CR (36% vs. 8%) [31].

## 3. Resistance to CAR-T Therapy in CLL and Attempts to Overcome It

The average CR in the studies listed in Table 1 is around 30%, ranging from 0% to 67%. Nevertheless, CAR T efficiency in CLL is low compared to other B cell malignancies such as acute B-lymphoblastic leukemia [13] and B-cell lymphoma [14,15,16]. Considering that only every third CLL patient achieves CR, it is crucial to find reasons for low efficiency of CAR T cell therapy in CLL. One explanation lies in T cell dysfunction in CLL patients. Defective immunological synapse formation of both CD4+ and CD8+ T cells with antigen presenting cells, including CLL cells, has been reported in CLL. Incomplete immune synapse formation has been linked to altered gene expression in T cells including the genes coding the molecules involved in actin polymerization, cytoskeletal organization and vesicle trafficking [36]. However, the same effect has been observed on allogenic T cells derived from healthy donors after contact with CLL cells [37]. It has been shown that circulating extracellular vesicles originating from CLL cells, which are abundant in CLL patients, induce exhaustion and attenuate CAR T cell function [38]. Defective immune synapse formation of CLL and both autologous and allogeneic T cells could be the consequence of the higher expression of B7- and TNF- receptor families of inhibitory transmembrane molecules [39]. Treatment of autologous T cells with the immunomodulating drug lenalidomide resulted in improved synapse formation [13,40], suggesting the therapeutic potential of a CAR T and lenalidomide combination strategy in CLL. The functional condition of T lymphocytes in CLL is described as exhaustion, due to the persistent antigenic stimulation with tumor antigens and consequent loss of their effector functions [41]. The tumor microenvironment in CLL is composed of tumor associated macrophages, myeloid derived suppressor cells, cancer associated fibroblasts, and nurse like cells (M2 macrophages) which create immunosuppressive environment and support maintenance of CLL cells [42]. However, nurse like cells and cancer associated fibroblasts probably contribute to CAR T cell failure in CLL [43,44]. These exhausted T lymphocytes in CLL patients highly express several transmembrane inhibitory receptors, including CTLA-4, PD-1 and LAG-3 [45,46]. It has also been reported that CLL patients with progressive disease have higher numbers of PD-1 and CTLA-4 expressing CD4+ and CD8+ lymphocytes compared to healthy controls [47,48]. These findings suggested that immune checkpoint inhibitors could have an important role in CLL treatment. Interestingly, it has been found that treatment with Bruton tyrosine kinase inhibitor (BTK), ibrutinib, decreases expression of the PD-1 on T cells and significantly increases expansion of CAR T cells in vivo [49]. In correlation with these results Gill et al. showed ORR in 71% and CR in 43% of patients who had not achieved CR despite 6 months of prior ibrutinib therapy and received anti-CD19 CAR T cells following standard lymphodepletion [32]. Gauthier et al. reported similar results in 19 CLL patients treated with anti-CD19 CAR T cells after ibrutinib failure [50]. In this study, patients had been treated with ibrutinib at least two weeks prior to leukapheresis and continued with ibrutinib for three and even more months after CAR T cell infusion. Overall response rate was 83% in CLL patients who received ibrutinib prior to CAR T cells therapy compared to 56% in patients who received CAR T cells without ibrutinib. Eighty-five percent of patients in the ibrutinib group achieved CR in bone marrow, compared to 50% in the group without ibrutinib [50].

**Figure 3:**
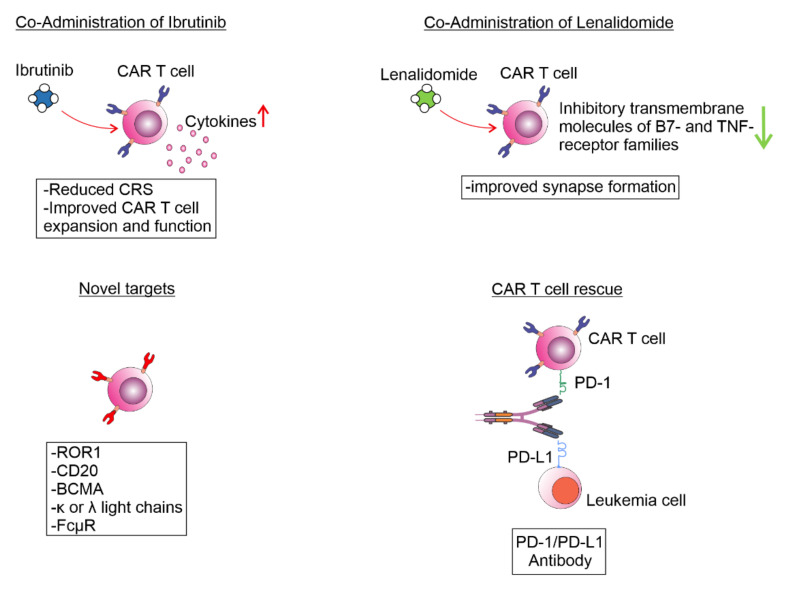
Novel attempts to improve CAR T cell therapy in CLL.

Although CD19 is a promising CAR T cells target, one likely mechanism of resistance to CAR T cells therapy is the loss of the target antigen [51]. Mutations of the CD19 coding gene and alternative splicing of CD19 mRNA [52,53] are some of the known mechanisms that contribute to antigen escape and relapse in acute lymphoblast leukemia, and possibly can contribute to a poor response to CAR T therapy in CLL. Several potential target antigens, besides CD19, have been investigated. These include anti-CD20 CAR T cells, tested on non-Hodgkin lymphomas with promising results [54]. Three patients received the third generation anti-CD20 CAR T cells, two patients without evaluable disease remained progression-free for 12 and 24 months and the third patient had an objective partial remission and relapsed at 12 months after infusions [54]. One ongoing study using anti-CD20 CAR T cells on relapsed/refractory B-cell malignancy including CLL (NCT03277729) is still in progress. Due to long-term B cell aplasia and hypogammaglobulinemia following anti-CD19 CAR T therapy, some studies focused on antigens that are highly expressed on malignant B lymphocytes but with low or without expression on normal B cells. Considering that CLL cells are monoclonal, they express only one type of light chain, κ or λ [55]. Different clones of normal B lymphocytes express monoclonal immunoglobulins with either κ or λ light chains implicating that CAR T cells targeting one type of light chain could partially spare normal B cells [10]. Results of one study that monitored the use of anti-κ light chain CAR T cells were modest, probably due to the lack of lymphodepletion regimen before CAR T therapy [31]. One possible target antigen highly expressed on CLL cells but without expression in normal mature B cells is a receptor tyrosine kinase-like orphan receptor 1 (ROR1) [56]. There are promising in vitro data [10] and one ongoing trial for the evaluation of anti-ROR1 CAR T therapy in ROR1+ malignancy including CLL patients (NCT02706392). Another selective target antigen for malignant B cells which can be used in CAR T therapy is the Fc receptor for immunoglobulin M (FcμR). FcμR is highly expressed by CLL cells and only minor levels are detected in healthy B cells. Anti-FcμR CAR-T cells from CLL patients purged their autologous CLL cells in vitro without reducing the number of healthy B cells [57]. These novel approaches to improve CAR T cell therapy in CLL are summarized in Figure 3.

## 4. Allogenic CAR T or CAR NK Cells in CLL Therapy?

Autologous CAR T cells therapy for CLL is promising, but there are several difficulties. Major problems are inability to collect enough viable T cells from CLL patients due to chemotherapy pretreatment and T cell dysfunction seen in CLL patient [15,39]. Another problem is long manufacturing time and delayed therapy. Despite promising results, CAR T cells therapy is linked to serious side effects such as cytokine-release syndrome grade 3 or 4 as a consequence of massive T cells activation and secretion of pro-inflammatory cytokines in large amounts. Immune effector cell associated neurotoxicity syndrome (ICANS) is also a potentially serious side effect of CAR T cells therapy [58].

Allogenic CAR T cells as a premanufactured product could be solution for previously listed problems. These cells are derived from a healthy donor’s lymphocytes. Using healthy donors provides high numbers of cells and peripheral blood mononuclear cells are fit, as donors, in contrast to cancer patients, who do not receive chemo- or radiotherapy [59]. Other cell sources for allogeneic CAR T cell development are umbilical cord blood-derived T cells. GVHD frequency and intensity can be decreased when using T cells obtained from umbilical cord blood, as these have reduced reactivity due to lower activation of the NF-κB pathway, resulting in decreased production of several pro-inflammatory cytokines [60,61]. Major advantages of allogenic CAR T cells are high antitumor potency and the ability to apply them immediately, without long manufacturing time. So far, allogenic CAR T cells have been used only in patients with B-cell malignancy, including five patients with CLL, who relapsed after allogenic stem cell transplant. Only one of these CLL patients achieved CR [25]. There are some trials using allogenic CAR T cells in acute lymphoblastic leukemia. UCART19 is a universal anti-CD19 CAR T-cell product that has been generated by simultaneously knocking out TCR and CD52, with the introduction of a CD19 directed CAR. Deletion of TCR and CD52 was performed to reduce the risk of UCART19 rejection [62]. The ORR was 67% in a phase 1 clinical trial of UCART19 in 21 patients with relapsed/refractory B-cell acute lymphoblastic leukemia [63]. UCART19 showed great results in children with relapsed/refractory acute lymphoblastic leukemia; two infants achieved complete molecular remission after lymphodepleting chemotherapy followed by a single-dose infusion of UCART19 cells [64]. Similarly, genetically modified allogenic CAR T cell product, PBCAR0191, achieved a CR rate of 71% in patients with relapsed/refractory diffuse large B cell lymphoma who received enhanced lymphodepletion prior to CAR T therapy [65]. Hence, allogenic CAR T cells from healthy donors could be a promising therapeutic strategy, although there are some concerns. Allogenic T cells could be activated by a recipient’s human leukocyte antigens (HLA) and in the state of the recipient’s immunosuppression can cause serious GvHD [66].

Since Natural Killer (NK) cells do not participate in immune rejection reactions, a new kind of allogenic CAR cells has been constructed—CAR NK cells [12]. These cells have a natural ability to kill malignant and infected cells without prior activation by antigen presenting cells and HLA restriction [67]. Problems with NK cells lie in poor expansion, difficulties in viral transduction and a shorter lifespan than T lymphocytes [68], but some authors achieved impressive results by co-culturing NK cells with modified feeder cells or activation beads [69,70] (Table 2).

CAR used for engineering CAR NK cells consists of an antigen-recognition domain and activation domains, similar to CAR T cells. There are four CAR NK generations, depending on CAR construction. The first generation of CAR NK cells contains only one activation domain, CD3 zeta, DAP10, or DAP12. The second generation includes one co-stimulatory domain (CD28, 4-1BB or 2B4) in tandem with CD3 zeta, or DAP10, or DAP12, while the third generation of CAR NK contains two co-stimulatory domains in the same tandem. The fourth CAR NK cells generation has been engineered to produce cytokine, the same as the fourth CAR T cells generation [67].

NK cells can be obtained from different sources—adult blood, cord blood, human induced pluripotent stem cells (hiPSCs) or human embryonic stem cells (hESCs) [71,72]. Some studies demonstrated higher activity and better expansion in vivo of cord blood CAR NK cells in comparison to CAR NK derived from adult blood [71,73]. These cord blood CART NK cells are currently being used in several clinical trials for the treatment of relapse/refractory CLL patients among other hematology malignancies (NCT03056339, NCT04796675).

In the last few years, hiPSCs have become the most promising source of NK cells. HiPSC-NK cells can be manufactured from standardized cells resulting in a homogeneous NK cell population [67]. These cells are perfect candidates for CAR NK manufacturing. IPSC-derived CAR NK cells known as FT596 have been used in a phase I clinical trial (NCT04245722) along with anti-CD20 monoclonal antibodies for relapsed/refractory B cell lymphoma. In the second and third single-dose cohorts of the monotherapy and combination arms comprising of a total of 14 patients, 10 of 14 patients (71%) achieved an objective response, including seven patients (50%) that achieved a complete response.

Nowadays, indications for CAR NK cells therapy in CLL remain an open question. CAR NK cells will probably be a good choice for patients without enough viable T cells for CAR T therapy and patients with aggressive disease who need early treatment. Additionally, for those patients that have relapsed after CAR T therapy or patients who develop high toxicity after CAR T infusion CAR NK, therapy may be a wise choice.

## 5. Conclusions and Future Perspective

CAR T cell therapy is promising for relapse/refractory CLL patients. Complete and durable remission of CLL is possible in patients treated with CAR T cells but further investigations are necessary to understand and possibly predict how patient specific factors influence the outcome of this treatment. It is also crucial to find ways to modulate recipients’ intrinsic immune milieu and enhance the efficiency of CAR T therapy. Concurrent use of ibrutinib with CAR T cells is promising. Despite high efficiency of CAR T therapy, there are major concerns related to debatable viability of CAR T cells derived from CLL patient and prolonged manufacturing time. CAR NK cells or allogeneic CAR T-cells with knocked out key genes associated with GvDH and immune rejection are maybe the answer for these problems. CAR NK can be derived from healthy donors and used as off-the-shelf product without prolonged manufacturing time.

## Figures and Tables

**Table 1:** CAR T cells trials in CLL. Abbreviation: ORR—overall response rate, CR—complete response, CLL—chronic lymphocytic leukemia.

Study (Reff. Number)	Number of CLL Patients	Target Antigen	Costimulatory Domain	ORR (%)	CR (%)
CAR-T					
[23]	8	CD19	CD28	0	0
[27]	3	CD19	4–1BB	100	67
[28]	4	CD19	CD28	75	25
[24]	4	CD19	CD28	25	0
[29]	5	CD19	CD28	100	60
[30]	14	CD19	4–1BB	57	29
[25]	5	CD19	CD28	40	20
[26]	2	IgKappa	CD28	0	0
[31]	32	CD19	4–1BB	44	28
[13]	24	CD19	4–1BB	71	17
[22]	8	CD19	CD28	75	25
[32]	19	CD19	4–1BB	53	53
[33]	10	CD19	4–1BB	60	40
[34]	19	CD19	4–1BB	79	21
[35]	3	CD19	4–1BB	100	67

**Table 2:** Comparison of allogenic CAR T and CAR NK cellsn.

	Allogenic CAR T Cells	CAR NK Cells
short manufacturing time	X	X
graft versus host reaction	X	
poor expansion in vitro		X
difficult viral transduction		X
long lifespan in vivo	X	
